# Visual tool for real-time monitoring of membrane fouling via Raman spectroscopy and process model based on principal component analysis

**DOI:** 10.1038/s41598-018-29268-y

**Published:** 2018-07-23

**Authors:** Tiina Virtanen, Satu-Pia Reinikainen, Jussi Lahti, Mika Mänttäri, Mari Kallioinen

**Affiliations:** Lappeenranta University of Technology, LUT School of Engineering Science, P.O. Box 20, 53851 Lappeenranta, Finland

## Abstract

Membrane fouling, *i.e.* accumulation of unwanted material on the surface of the membrane is a significant problem in filtration processes since it commonly degrades membrane performance and increases operating costs. Therefore, the advantages of early stage monitoring and control of fouling are widely recognized. In this work, the potential of using Raman spectroscopy coupled to chemometrics in order to quantify degree of membrane fouling in real-time was investigated. The Raman data set collected from adsorption experiments with varying pHs and concentrations of model compound vanillin was used to develop a predictive model based on principal component analysis (PCA) for the quantification of the vanillin adsorbed on the membrane. The correspondence between the predicted concentrations based on the PCA model and actual measured concentrations of adsorbed vanillin was moderately good. The model developed was successful in monitoring both adsorption and desorption processes. Furthermore, the model was able to detect abnormally proceeding experiment based on differentiating PCA score and loading values. The results indicated that the presented approach of using Raman spectroscopy combined with a PCA model has potential for use in monitoring and control of fouling and cleaning in membrane processes.

## Introduction

Membrane based technologies have been widely applied for purification, concentration and separation of fluids ranging from surface water and wastewater to a variety of industrial streams^1–3^. The performance of membrane processes is strongly hampered by membrane fouling, which typically leads to decline of production volume and can even change the rejection characteristics of the membrane^3–5^. Membrane fouling also leads to the need to clean or replace the membranes, which increases operational costs^6,7^. The fouling problem can be mitigated by adoption of fixed operational schemes consisting of membrane aeration, backwash and chemical cleaning cycles. However, such conservative anti-fouling strategies ignore the dynamic nature of fouling and are therefore never optimal^8–10^. Thus real-time early warning systems for monitoring of fouling and optimized dynamic fouling control schemes that are able to minimize the build up of a foulant layer are needed to improve the operation of membrane processes^11–14^. Process analytical technologies (PAT) enable continuous analysis and thus can be applied for learning and quality control of processes on the basis of real-time monitoring^15^. In this work, a chemometric approach based on online Raman spectroscopy is taken.

Since short sampling intervals are needed to follow membrane processes in real time, the acquired data sets are usually large. However, the patterns in the spectral data can be easily modeled, visualized and reduced to more easily interpretable form using chemometric methods. They offer a valuable for toolbox for building an empirical model of the desired process behavior^15–17^. Principal component analysis (PCA) is the most simple multivariate chemometric method which is widely used for compression and information extraction from large data sets^18^. PCA can be utilized in the interpretation and classification of large data sets and in outlier detection^19^. PCA captures a set of underlying variables that are uncorrelated with each other. These new variables, *i.e*. principal components (PCs) describe major trends in the original data set^20^. Simple and linear dimensionality reduction can be done also with another popular multivariate method called partial least squares regression (PLS)^18^.

Spectroscopic techniques coupled with multivariate chemometric models have proven to be promising techniques for quantitative and qualitative evaluation of membrane filtration processes. Lyndgaard *et al*.^21^ demonstrated that ultraviolet (UV) spectroscopy in combination with principal component analysis (PCA) and partial least squares (PLS) modeling can be used as a method for monitoring cleaning of whey filtration membrane units. UV data was also utilized by Corona *et al*.^22^ to develop various multivariate regression models to estimate the nitrate-nitrogen concentrations in a denitrifying post-filtration unit of a wastewater treatment plant. Skou *et al*.^23^ combined near infrared spectroscopy (NIRS) with PLS regression modeling to monitor quality of a reverse osmosis polisher filtration unit permeate that was sought to be reused as process water in dairy industry. In addition, Elshereef *et al*.^24^ developed multivariate regression models for monitoring the changes in concentrations of proteins in both permeate and retentate of a whey protein isolate filtration by making use of data collected by fluorescence spectroscopy.

Raman spectroscopy is able to capture information about the quality and quantity of molecules based on their vibrational transitions and is therefore a suitable method for elucidating various chemical processes^25^. Raman based spectroscopic techniques combined with multivariate modeling has been recently applied *e.g*. for quantification of strong acid concentration in solutions^26^, to monitor lignocellulosic bioethanol production processes^27^ and for predictive modeling of cell culture growth and metabolite production^28^. Our previous studies have shown that online measurement by Raman spectroscopy can be used for rapid, direct and non-invasive detection of adsorption processes on the surface of the membrane^11,29^. However, in our previously published approach the chemometric analysis run was done individually for each adsorption experiment data set and scaling of the results for getting quantitative accumulation curves required *ex situ* calibration measurements. Thus direct real-time quantitative estimation of membrane fouling based on only Raman spectral data and chemometric model has not been presented earlier.

**Figure 1:**
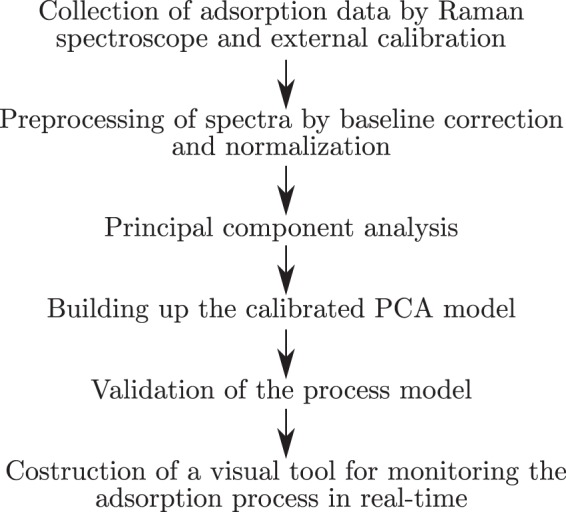
Summary of the procedure for Raman and PCA based membrane process monitoring and control technique.

In this study, we propose the application of Raman spectroscopy with PCA to build a chemometric calibration model that allows prediction of unknown concentrations and visualization of development of adsorptive fouling on the surface of the membrane in real-time^30^. Membrane fouling caused by phenolic and ligneous compounds is a significant problem in the membrane filtration field and understanding on the profound fouling mechanisms is needed. Thus vanillin was used as a adsorptive model compound due to its lignin-related structure, phenolic amphipathic nature and intense Raman response of the delocalized π-electrons. Previously acquired Raman spectra and external calibration data^11,29^ were used to build a PCA model describing a smoothly running membrane process. Validation of the method was performed through a comparison of measured values to those predicted by the model. The correspondence between the predicted concentrations and actual measured concentrations of adsorbed vanillin was fairly good. Thus the proposed approach has potential for use in visual monitoring of adsorption and desorption during membrane processes and for differentiation of anomalous events based on score and loading values. In addition the model has a potential to be used as a part of a process control system to reach the desired concentration level of the monitored compound either in an adsorptive coating process or in monitoring the effect of a washing process. The suggested procedure for Raman spectroscopy and PCA based monitoring and control is presented in Fig. 1.

## Results

### Spectral changes revealed by PCA loadings

When the number of wavelenghts is reduced to the ones that carry most information, the model is typically safer and easier to interpret and the prediction ability might be increased^31^. In the spectral area used in this study the peaks in the spectrum of the polyethersulfone membrane at 1580 cm^−1^ and 1600 cm^−1^ originate from stretchings of the aromatic ring^32^. The Raman spectrum of the vanillin powder has peaks at 1511 cm^−1^ and at 1592 cm^−1^ (coupled skeletal stretchings of the aromatic ring) and peak with a shoulder at 1665 cm^−1^ (C=O stretching of the aldehyde group)^33^. By analyzing information contained in Raman spectra, it is possible to capture spectral changes that are related to chemical changes on the surface of the membrane. PCA analysis compresses data into loading and score values of main principal components that describe most of the variation in the data.

**Figure 2:**
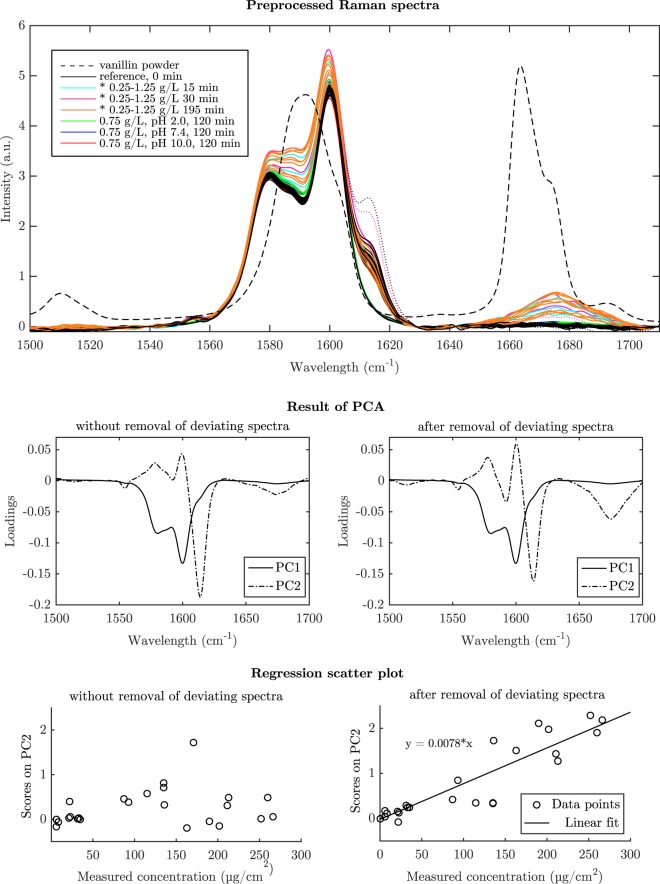
Preprocessed Raman spectra show how the intensity of vanillin peaks on the membrane increase as the concentration of the vanillin increases (*0.25 g/L = pH 5.5, 0, 75 g/L = pH 5.10 and 1.25 g/L = pH 5.0). Spectra were sent to the PCA analysis with and without removing deviating spectra which possessed a fairly intense shoulder peak at 1615 cm^−1^ (dotted lines). It can be seen that anomalous spectra with intense signals in the studied region may skew the result of the PCA model significantly to overemphasize the spectral changes in that region. This results in incorrect scores values.

By examining the loadings related to each PC, it is possible to identify which deviations in the spectral variables are explained by a given PC^34^. Since spectra were not mean centered prior to PCA, the loadings of PC1 represent the average spectrum and PC2 represents changes to the average spectrum^25^. The loading plot of PC1 represents the spectrum of the membrane and contains a major peak at 1600 cm^−1^ and a minor peak in the form of a shoulder around 1580 cm^−1^ (Fig. 2). The presence of these loading peaks of PC2 at similar locations as the Raman peaks of vanillin indicate that PC1 is mostly correlated with the vanillin content.

The original calibration data set yielded the loading plot shown in Fig. 2. Two spectra were discarded from the calibration model due to strong, anomalous shoulder peaks at 1615 cm^−1^ (Fig. 2). It can be seen how significantly deviating spectra can change acquired loadings by emphasizing the area where the deviation is present. This substantive change in model structure can be explained by the small size of the data set. When spectral data is studied, the outliers are typically easily detected because spectra are usually very accurate measurements. Generally when analyzing large data sets the PCA focuses more on main trends instead of on noise and singular changes. As shown also here, very often removal of a serious outlier results in significant changes in the model. If the outlier is not removed the model focuses on modeling errors instead of interesting variations^31,35^.

### Construction of calibrated PCA model based on score values

After the Raman spectra have been subjected to spectral preprocessing and PCA analysis the obtained score values and external calibration data are used to build a PCA model that presents the adsorption process. Score values describe how the variation in the data changes between different measurements. In this study development of score values as a function of time describes how intensities of vanillin peaks are increasing as it accumulates on the membrane. Intensities of the peaks increase with increasing concentration of the vanillin. Hence spectra with high vanillin concentration are associated with high PC1 scores.

**Figure 3:**
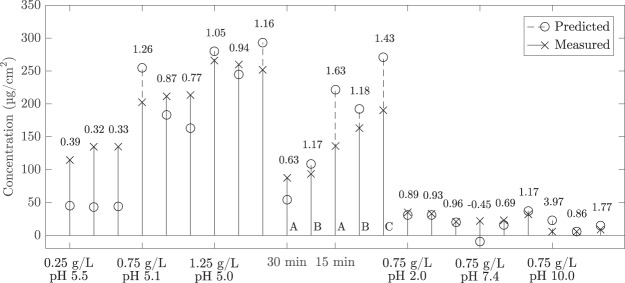
Predicted concentrations of the adsorbed vanillin plotted against measured concentrations for the test set used in the calibration. The multiplier needed to get the measured value from the estimated value is shown over each stem. A = 0.25 g/L, B = 0.75 g/L and C = 1.25 g/L.

With the available external calibration data from extractions and UV analysis, it is possible to make a plain regression model between Raman spectra and concentration of adsorbed vanillin. It can be assumed that in the beginning of the adsorption measurement the concentration of vanillin on the surface of the membrane is 0. The concentration of the vanillin in the end of the experiment can be picked up from extraction data. The extension of the Beer-Lambert law gives a dependency between intensity of Raman scattering and concentration. The equation for linear regression is:

1 $$c=ks,$$

where *c* is the measured concentration and *s* is a scaled scores value, which represents the intensity of the vanillin peak in the spectrum. Scaling was done subtracting the reference score values (*t* = 0 min) of each experiment both from the initial reference score value and from the final score value (*t* = *i* min). Regression coefficient *k* relates the score value and the measured concentration. The model can be tested simply by looking at a plot of calculated against measured data. Figure 2 shows the deviation between predicted and measured results for the test set. In an ideal case all data points should be on the diagonal, but some spread is common. The regression coefficient (*k* = 0.0079) can be used on the test data to predict the concentration levels during adsorption or desorption processes. Even though the match is not perfect, the main trends can be observed in the predicted data. If the deviation is not too wide, the predicted values might be good enough for detecting the level of concentration and to tune the process^30^. A comparison of predicted and measured concentrations is shown in Fig. 3. It can be seen that the model can either over- or underestimate the concentration depending on the case. The multiplier needed to get the measured value from the estimated value is in the best case 0.96 and in the worst case 3.97.

Predicted and measured vanillin concentrations are far apart *e.g*. at 0.25 g/L and pH 5.5. As the distribution of data points is somewhat uneven and in the other end of the diagonal centered around the lower concentrations PCA model seems to be more effective in that region than in the middle of the diagonal. At the other end extreme data points with higher variation might span the model to focus more on the higher concentrations and the accuracy of the middle region might be hampered also due to that. In the ideal case dataset should cover the studied range as representatively as possible and a better model would cover wider area of variations in the data and result score plot where the sample points are more evenly spread over the whole diagonal. Problems in the prediction might also stem from combined sampling and analytical uncertainty due to uneven concentration profile, modeling errors and from noise in the data^31^.

### Monitoring based on the calibrated PCA model

Visualization of data is an essential part of chemometric data analysis. Principal components can be presented either by line or scatter plots^30^. Our example from membrane process monitoring shows how online measurement by normal Raman spectroscopy can be used for fast prediction of concentration of adsorbed vanillin on the surface of the commercial polyethersulfone membrane. The previously built up PCA based model is used for this purpose.

**Figure 4:**
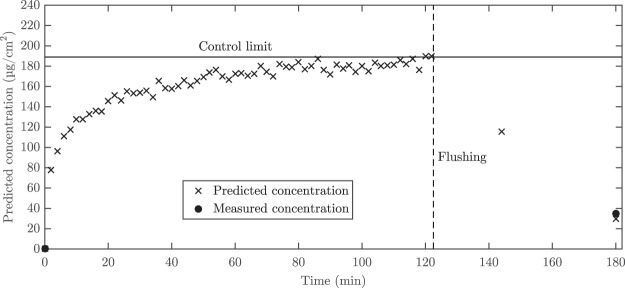
An example control chart based on the PCA model. The predicted concentrations for data from experiment 1/3 of 0.75 g/L at pH 2.0 are shown as an example.

Data from experiment 1/3 of 0.75 g/L at pH 2.0 was treated as an example. PCA was first run for the initial spectrum, one by one for each adsorption spectrum and for calibration data (all initial and final spectra). The run was done separately for each adsorption time point. The initial spectrum was included in all runs to enable scaling. The acquired PC2 score values for the initial spectrum and adsorption spectrum were scaled by subtracting the score value of the initial spectrum from score values of both spectra. The scaled score values were 0 for initial spectra and between −0.61 and −1.48 for adsorption spectra. Then the score values were multiplied by −1 to get positive scores and divided by the correlation coefficient *k* = 0.0078 (from Fig. 2) to gain the predicted concentrations. The scatter plot for predicted concentrations is shown in Fig. 4.

The score value that represents the current state of the process can also be used as a criterion for ending the process or for another control actions such as flushing of the membrane. This is enabled by multivariate control charts, which can classify if samples are “in” and “out” of control based on the determined score values and subsequent concentration levels^36^. This approach is demonstrated by setting the arbitrary “control limit” to the chart in Fig. 4. The chart shows how the predicted data points can be easily classified as “in control” (concentrations within the normal operating range) or “out-of-control” based on the acquired scores values and predicted concentrations.

## Discussion

In this study, a process model based on Raman spectroscopy and PCA was shown to posses monitoring capability for adsorption processes on the surface of membranes. Comparison of estimated and *ex situ* measured results showed fairly good correspondence between the predicted concentrations based on the PCA model and actual measured concentrations of adsorbed vanillin. When compared to conventional offline analytical laboratory methods, it is usual that some accuracy has to be sacrificed to achieve real-time results^37^. Thus the presented procedure has potential to serve as a tool for development of membrane fouling control strategies and process optimization. A well built model could be routinely applied to online data in order to predict the development of the concentrations of the adsorbed phenolic foulants.

The accuracy of the predictions could be improved by using larger data set for building the model. In generally for the process analytical application, the model building and testing are the most important aspects^37^. The presented approach, which is based on the PC scores generated by PCA of Raman spectra, is believed to be suitable for real-time monitoring of membrane fouling and control/optimization of membrane systems. Raman spectroscopy is able to detect chemical changes caused by the foulants on the surface of the membrane rapidly and in non-invasive way. Then PCA can be utilized to distinguish, classify and visualize changes in the spectral data. In addition, score values produced by PCA can be used as a criterion for process control actions. The advantage of a PCA based monitoring approach is that the generated monitoring charts are simple and easily interpretable.

Due to dynamic nature of membrane processes the principle of the updating monitoring schemes should be in practice that when new spectra are available they are included in the PCA model data matrix according to certain weights. However, because in dynamic processes data are autocorrelated, *i.e*. each observation is dependent on the previous observation, care should be taken to ensure that deviating spectra do not skew the monitoring output results too significantly^17^. Finding outliers which are atypical objects or variables is crucial for all the chemometric methods and they must be either removed or pre-treated to gain robust models. It is essential to keep outliers only when they represent some important property in the data^31^. This study demonstrates how only one spectrum with anomalous and moderately intense peak in the analyzed region can overemphasize the spectral changes and yield distorted PCA model. However, thanks to this tracking of the occurrence of observable perturbations is possible in some cases.

All in all the combination of spectroscopy and and chemometrics is a potential solution for fast and nondestructive analysis of the studied adsorption process. Economic benefits of chemometric process analysis include comparatively small capital requirements in sensor and computer time, safer process operation and better understanding of a process due to the flow of real-time information. A dynamic control system may also improve quality of product due to maintenance of tighter control limits, save energy, increase membrane life-time, minimize waste and improve production capacity through process optimization.

## Methods

### Real-time fouling monitoring using normal Raman spectroscopy

The Raman data collection process used has been described in detail in previous studies^11,29^. Raman spectra were recorded during cross-flow experiments using a Kaiser RXN1 spectrometer with 785 nm laser excitation and maximum power of 400 mW. Spectra were collected between 100–3425 cm^−1^ using immersion optics (0 mm focal length) with an MR probe head, which was placed above the surface of the hydrophilic polyethersulfone membrane (UH004P supplied by Microdyn-Nadir GmbH).

Raman spectra were obtained from two different experimental sets. In the first experimental set^11^ adsorption data was collected using varying concentrations of vanillin (0.25, 0.75 and 1.25 g/L). pH values of the vanillin solutions were 5.5, 5.1 and 5.0 respectively. Different adsorption times (15, 30 and 195 min) were also tested. In the second experimental set^29^ the concentration of vanillin (0.75 g/L) and adsorption time (120 min) were constant but the pH was varied (pH 2, 7.4 and 10). Tests were repeated three times for each conditions. The short 15 min and 30 min adsorption experiments were done only once in each concentration.

The raw Raman spectra used are shown in Fig. S1 (Supplementary Information). The spectra are highly overlapping and the baseline and intensity levels drift between different experiments. Hence visual inspection permits only main features of the spectra to be distinguished. It can be observed that adsorption of vanillin results in the appearance of new peaks.

### Preprocessing of spectral data

In the ideal case, to capture all the variability and changes caused by membrane fouling, the full spectra should be analyzed as opposed to individual peaks^20,34,38^. However, because the accuracy of the model is usually weak for full spectra, from the practical point of view it is better to take only a small and relevant spectral area into analysis^19^. Since the raw spectra are affected by noise, overlapping and spectral drift, preprocessing methods need to be applied before running PCA.

Before performing PCA analysis, spectra were baseline corrected^39^ and normalized by setting the intensity of the peak between 1070–1075 cm^−1^ to the value 1. All data processing was performed using MATLAB (R2016a).

### External calibration data from extractions and UV analysis

The extraction method and UV analytical techniques for acquiring external calibration data used here have been published in detail in previous studies^11,29^. Briefly, the vanillin adsorbed on the membranes was extracted using methanol and UV absorptions of the extracts were measured using a Jasco V-670 spectrophotometer at the wavelength of 308 nm.

### Principal component analysis

Principal component analysis (PCA) is able to analyze variation in large spectral data sets over their whole wavelength range. In previous studies^11,29^, PCA was successfully applied to analyze, how vanillin accumulates on the surface of the commercial polyethersulfone membrane UH004 P (Microdyn-Nadir GmbH) as a function of time.

In this study, PCA was performed on a limited area of the spectrum at 1500–1700 cm^−1^. The initial data matrix of spectral data contained 48 rows (different experiments) and 202 columns (intensities of the corresponding Raman spectra). PCA decomposes the data matrix $X$ as the sum of the outer product of vectors ${{\boldsymbol{s}}}_{{\boldsymbol{i}}}$ (scores) and ${{\boldsymbol{p}}}_{{\boldsymbol{i}}}$ (loadings) plus a residual matrix $E$ as presented in the following equation:

2 $$X=\sum _{i=1}^{n}{{\boldsymbol{s}}}_{{\boldsymbol{i}}}\cdot {{\boldsymbol{p}}}_{{\boldsymbol{i}}}+E,$$

where *n* is the number of samples in the *X* data set. Scores represent new variables with compressed information extracted by PCA. Loadings contain information on how the variables are correlated. The performed PCA resulted in two PCs, which captured 99.76% of the total variance present in the data. The remaining variance (0.24%) was due to noise in the data.

### Data availability

The data sets analyzed during the current study are available from the corresponding author on reasonable request.

## Electronic supplementary material

Supplementary Information
