# Supplementary material for: Visual tool for real-time monitoring of membrane fouling via Raman spectroscopy and process model based on principal component analysis
Source: Sci Rep. 2018 Jul 23;8:11057. doi: 10.1038/s41598-018-29268-y (PMC6056556; doi:10.1038/s41598-018-29268-y)
Supplement: Supplementary file 1 — Supplementary Information [file 41598_2018_29268_MOESM1_ESM.pdf]

Supplementary Information

Visual tool for real-time monitoring of membrane fouling via Raman spectroscopy and process model based on principal component analysis

Tiina Virtanen[1,*], Satu-Pia Reinikainen[1], Jussi Lahti[1], Mika Mänttäri[1], and Mari Kallioinen[1]

[1]Lappeenranta University of Technology, LUT School of Engineering Science, P.O. Box 20, 53851, Lappeenranta, Finland
[*]tiina.virtanen@lut.fi

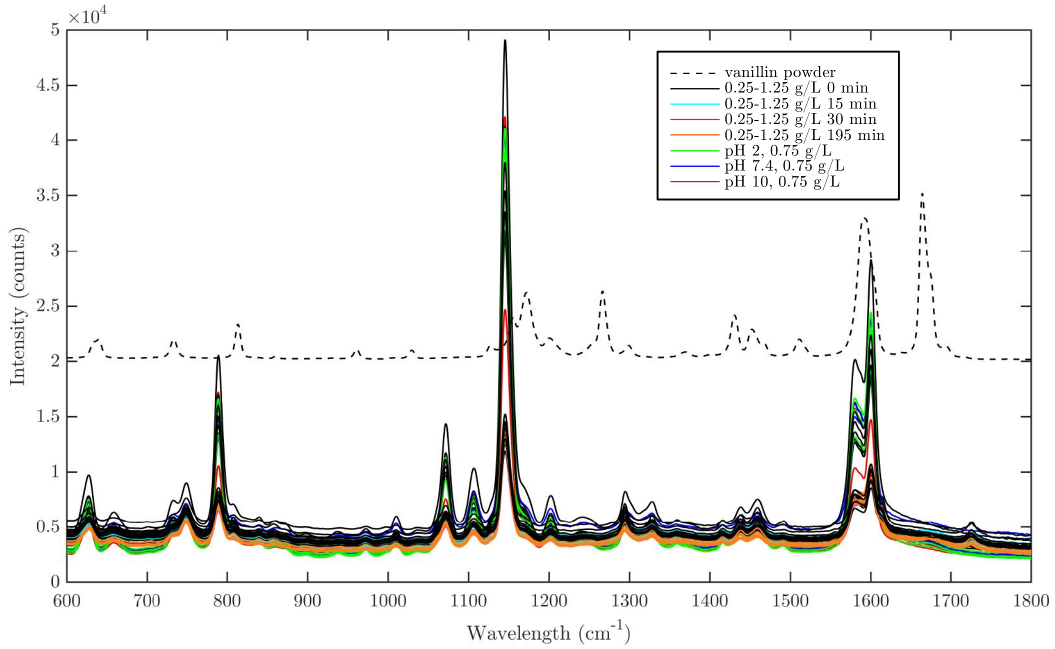

Figure S1: Raw Raman spectra.
